# Bipolar transurethral enucleation and resection of the prostate versus bipolar resection of the prostate for prostates larger than 60gr: A retrospective study at a single academic tertiary care center

**DOI:** 10.1590/S1677-5538.IBJU.2015.0225

**Published:** 2016

**Authors:** Yong Wei, Ning Xu, Shao-Hao Chen, Xiao-Dong Li, Qing-Shui Zheng, Yun-Zhi Lin, Xue-Yi Xue

**Affiliations:** 1 Department of Urology, the First Affiliated Hospital of Fujian Medical University, Fuzhou, China

**Keywords:** Prostatic Hyperplasia, Transurethral Resection of Prostate, Prostate

## Abstract

**Objective:**

To evaluate the efficacy and safety of bipolar transurethral enucleation and resection of the prostate (B-TUERP) versus bipolar transurethral resection of the prostate (B-TURP) in the treatment of prostates larger than 60g.

**Material and Methods:**

Clinical data for 270 BPH patients who underwent B-TUERP and 204 patients who underwent B-TURP for BPH from May 2007 to May 2013 at our center were retrospectively analyzed. Outcome measures included operative time, decreased hemoglobin level, total prostate specific antigen (TPSA), International Prostate Symptom Score (IPSS), maximal urinary flow rate (Qmax), quality of life (QoL) score, post void residual urine volume (RUV), bladder irrigation duration, hospital stay, and the weight of resected prostatic tissue. Other measures included perioperative complications including transurethral resection syndrome (TURS), hyponatremia, blood transfusion, bleeding requiring surgery, postoperative acute urinary retention, urine incontinence and urinary sepsis. Patients in both groups were followed for two years.

**Results:**

Compared with the B-TURP group, the B-TUERP group had shorter operative time, postoperative bladder irrigation duration and hospital stay, a greater amount of resected prostatic tissue, less postoperative hemoglobin decrease, better postoperative IPSS and Qmax, as well as lower incidences of hyponatremia, urinary sepsis, blood transfusion requirement, urine incontinence and reoperation (P<0.05 for all).

**Conclusions:**

B-TUERP is superior to B-TURP in the management of large volume BPH in terms of efficacy and safety, but this finding needs to be validated in further prospective, randomized, controlled studies.

## INTRODUCTION

Transurethral resection of the prostate (TURP) has long been considered the gold standard for treatment of symptomatic benign prostatic hyperplasia (BPH) when medical therapy fails (1, 2). Conventional TURP uses a monopolar electrocautery system in which distilled water or a variety of solutions other than normal saline are used as an irrigant (3). Although monopolar TURP has a high success rate (90%-95%), it is associated with a morbidity rate of 15% to 18% and a mortality rate of 0.001% (4). Bipolar TURP (BTURP), with the use of normal saline as irrigant, significantly eliminates the risk of transurethral resection syndrome (TURS) (3–5). B-TURP is associated with significantly less fluid absorption than monopolar TURP, but the operative duration and the weight of resected prostatic tissue are similar between the two procedures (6). In addition, postoperative bleeding, blood transfusion requirements, early and late complications such as clot retention, urinary retention, bladder neck stenosis and urethral stricture did not significantly differ between the two procedures (7–10). There is still a need to upgrade this technique to improve its efficacy and safety.

Transurethral enucleation and resection of the prostate (TUERP) is a recently developed procedure created by Liu et al. (11), in which the prostate is transurethrally enucleated and resected using a bipolar plasma kinetic resectoscope (12). Studies have suggested that TUERP is a safe and feasible treatment for BPH with few complications (12–15). Although several studies have demonstrated better clinical benefits for TUERP than for other treatments (13, 16), this procedure has not been widely accepted. This study aimed to compare the efficacy and safety of B-TUERP versus B-TURP in the management of prostates larger than 60g.

## MATERIAL AND METHODS

### Patients and study protocol

The study was approved by the Medical Ethics Committee of the First Affiliated Hospital of Fujian Medical University. All patients provided written informed consent. The clinical data for 298 consecutive patients who underwent BTUERP and 225 consecutive patients who underwent B-TURP for BPH from May 2007 to May 2013 at our center were retrospectively analyzed. All operations were performed mainly by one surgeon (Xue X-Y), who has more than twenty years of experience with these procedures. The type of operation was selected according to the patient's preference after detailed explanation by the surgeon regarding the procedures, outcomes, and complications of each option. All the patients had histologically proved BPH and only those with prostate volume larger than 60g on transrectal ultrasound were included (9, 17). Any patient with a previous history of prostatic or urethral surgery, urethral stricture, neurovesical dysfunction and/or prostate cancer was excluded. Indications for surgery were a preoperative International Prostate Symptom Score (IPSS) ≥12 points, a maximal urinary flow rate (Qmax) <15mL/s, urine retention, upper tract dilatation, renal insufficiency and recurrent urinary tract infection. B-TUERP or B-TURP was done according to patient's preference. Age, IPSS, quality of life (QoL) score, prostate specific antigen (PSA), prostatic volume (PV) and post-void residual urine volume (RUV) were compared preoperatively between the two groups. A total of 474 (90.6%) of 523 patients were followed for two years, and the others were lost to follow-up.

### Operative techniques

Both bipolar resection procedures were performed using the Gyrus bipolar plasmakinetic resection system, with the power set at 200W for cutting and at 100W for coagulation. Normal saline was used as irrigant, and the irrigation pressure ranged from 80 to 100mH_2_O. Cystostomy was not performed in all cases. Under general or spinal anesthesia, the patient was placed in the lithotomy position. A 27-Fr resectoscope was placed in the bladder under video assisted endosurgical system guidance.

B-TURP was performed as previously described (18). Transurethral resection of prostatic hyperplasia tissue was performed along the direction from the mouth of the urethra to the prostate apex and from the urethra to the prostatic capsule.

B-TUERP was conducted also as previously described (12). Briefly, an incision was created close to the verumontanum in order to incise the urethral mucosa deep to the level of the surgical capsule. After dissecting the distal mid lobe and mucosa in a retrograde fashion toward the bladder neck and detaching adenoma of the distal mid lobe from the surgical capsule, the denuded supply vessels and hemorrhage spots on the capsule surface were identified and coagulated to block the lobe blood supply. The bilateral lobes along the surgical capsule were then detached and all supply vessels were coagulated. The adenoma was finally resected. When resection was completed, all adenoma fragments were extracted using an Ellik evacuator, and a 20-F 3-way Foley catheter was placed and connected to straight drainage until hematuria sufficiently resolved.

### Outcome measures

Operative time, pre-and postoperative hemoglobin levels (on the first postoperative day), weight of resected prostatic tissue, bladder irrigation duration, hospital stay, IPSS, Qmax, QoL score, RUV, and TPSA were calculated. Perioperative complications such as TURS, hyponatremia (at the end of operation, defined as serum sodium less than 135mmol/L), blood transfusion, bleeding requiring surgery to stop bleeding, postoperative acute urinary retention, urine incontinence and urinary sepsis were observed.

### Follow-up

Patients in both groups were followed for two years. One independent investigator performed the follow-up at 1, 6, 12, and 24 months. Postoperative outcome measures, including Qmax, PSA, IPSS, RUV, and QoL score, were recorded at each follow-up visit. Urethral stricture, bladder neck stenosis, urine incontinence and postoperative acute urinary retention, as well as postoperative recurrence requiring reoperation were also recorded during the follow-up period.

#### Statistical analysis

Statistical analyses were performed using Statistical Package for the Social Sciences (SPSS Inc., Chicago, IL, USA). Data following a normal distribution are presented as mean±standard deviation and were compared using the t-test, while data not following a normal distribution are presented as median (range) and were compared using the Wilcoxon rank-sum test for two independent samples. Categorical data (percentages) were compared using the chi-square test or the Fisher's exact probability test. P-values <0.05 were considered statistically significant.

## RESULTS

### Baseline patient characteristics

The baseline characteristics of the included patients are shown in Table-1. There were 270 patients in the B-TUERP group and 204 patients in the B-TURP group. Preoperatively, the two groups had comparable mean age, IPSS, QoL score, TPSA, PV and RUV (P>0.05 for all).

**Table 1:** Baseline characteristics of the included patients.

	B-TUERP	B-TURP	P
No. of cases	270	204	-
Age (year)	68.0±8.6	68.4±7.9	0.588
IPSS	25.4±5.2	25.0 ± 5.7	0.431
QoL score	3.5±1.4	3.5 ± 1.6	0.806
Median preoperative TPSA (interquartile range)	3.70 (2.52-6.25)	3.67 (2.39-6.19)	0.748*
PV (mL)	80.1 ± 11.1	80.7 ± 12.5	0.578
Qmax (mL/s)	5.7±2.6	5.3±2.3	0.089
RUV (mL)	140.1±43.4	136.5±41.0	0.369

**B-TUERP** = bipolar transurethral enucleation and resection of the prostate; **B-TURP** = bipolar transurethral resection of the prostate; **IPSS** = International Prostate Symptom Score; **Qmax** = maximal urinary flow rate; **QoL** = quality of life; **TPSA** = total prostate specific antigen; **PV** = prostatic volume; **RUV** = residual urine volume.

* Mann-Whitney test.

### Perioperative and postoperative outcomes

All procedures were successful, and no conversion to open surgery was required. There were no perioperative cardiovascular or cerebrovascular accidents following the two procedures. Perioperative outcomes in the two groups are summarized in Table-2. The B-TUERP procedure required significantly shorter operative time than the B-TURP procedure (P<0.05). Postoperative hemoglobin decrease was more significant in the BTURP group compared with the B-TUERP group (P<0.05). The weight of resected prostatic tissue was greater in the B-TUERP group (P<0.05). In addition, postoperative bladder irrigation duration and hospital stay were significantly shorter in the B-TUERP group than in the B-TURP group (P<0.05 for both).

**Table 2:** Perioperative outcomes in the two groups.

	B-TUERP	B-TURP	P
Operative time (min)	73.37 ± 19.99	83.77 ± 20.89	<0.001
Hospital stay (d)	4.0	5.0	<0.001*
(interquartile range)	(4.0-5.0)	(5.0-6.0)	
Decreased hemoglobin (g/L)	1.79 ± 0.51	2.35 ± 0.63	<0.001
Postoperative bladder irrigation duration (h)	32.56 ± 8.97	58.92 ± 12.93	<0.001
Weight of resected prostatic tissue (g)	43.2±12.9	40.4±11.6	0.013

**B-TUERP** = bipolar transurethral enucleation and resection of the prostate; **B-TURP** = bipolar transurethral resection of the prostate.

* Mann-Whitney test.

**Figure 1:**
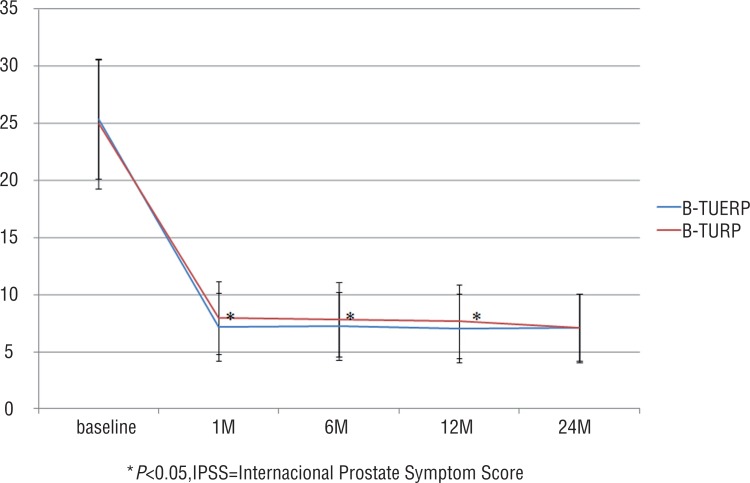
Mean IPSS scores before and after treatment.

**Figure 2:**
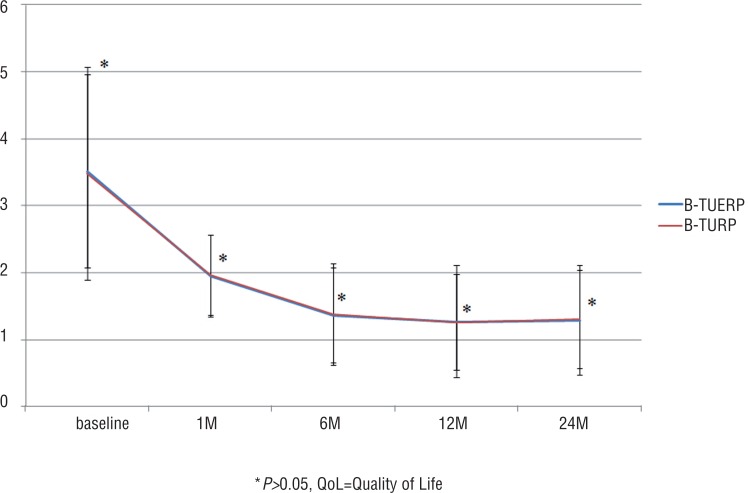
Mean QoL scores before and after treatment.

**Figure 3:**
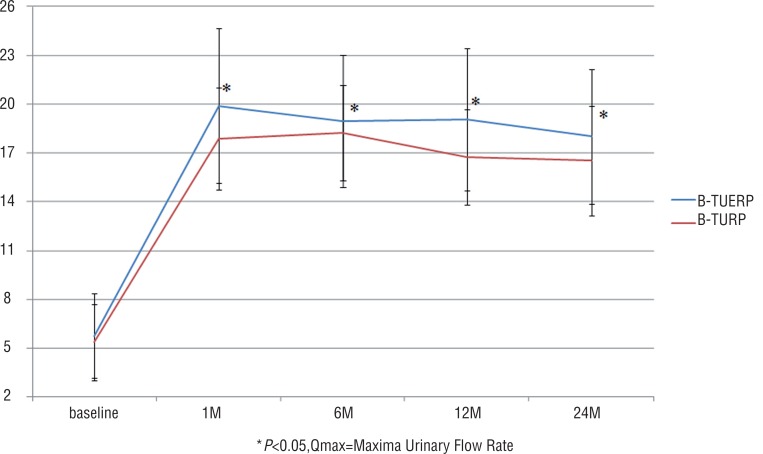
Mean Qmax scores before and after treatment.

**Figure 4:**
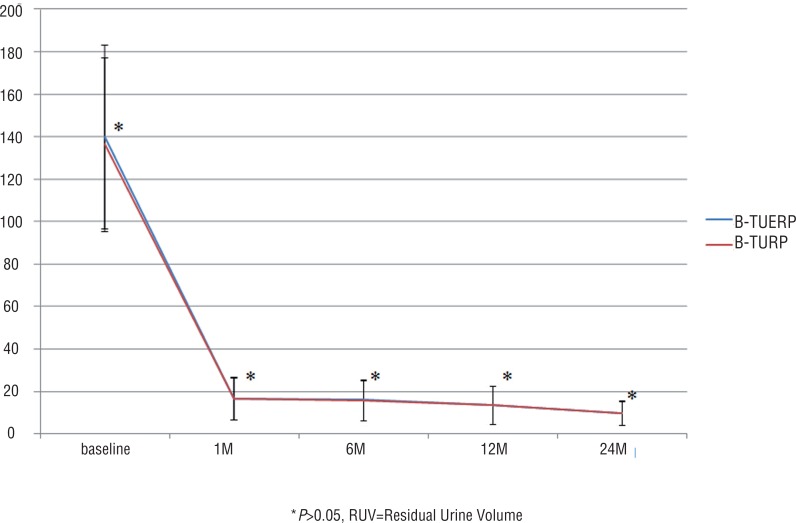
Mean RUV scores before and after treatment.

Postoperative QoL score and RUV at all follow-up time points were similar between the two groups (P>0.05 for all), but postoperative IPSS at 1, 6 and 12 months and Qmax at all follow-up time points were significantly better in the BTUERP group than in the B-TURP group (P<0.05 for both) (Figures 1–4).

### Perioperative and postoperative complications

Perioperative and postoperative complications in the two groups are presented in Table-3. No TURS occurred in either group. Six patients in the B-TURP group developed hyponatremia, while only two patients developed hyponatremia in the B-TUERP group (P<0.05). The number of patients requiring blood transfusion was significantly lower in the B-TUERP group than in the B-TURP group (P<0.05). At one month, urinary incontinence rate was significantly lower in the BTUERP group than in the B-TURP group (P<0.05), but this resolved within three months. However, there were no significant differences in the incidence of urethral stricture, bladder neck stenosis, bleeding requiring surgery or postoperative acute urinary retention.

**Table 3:** Complications of B-TUERP and B-TURP classified according to the modified Clavien system.

Complications	B-TUERP	B-TURP	P
Grade I, n(%)	19 (7.0%)	34(16.7%)	0.001
Hyponatremia, n(%)	2 (0.7%)	6(2.9%)	0.139
Postoperative urinary sepsis, n(%)	0 (0%)	4(2.0%)	0.071*
Postoperative acute urinary retention, n(%)	2 (0.7%)	2(1.0%)	1.000*
Bladder neck stenosis, n(%)	1(0.4%)	1(0.5%)	1.000*
Incontinence at 1 month, n(%)	14(5.0%)	21(10.3%)	0.035
Grade II, n(%)	0 (0%)	3(1.5%)	0.079*
Blood transfusion requirement, n(%)	0 (0%)	3(1.5%)	0.079*
Grade III, n(%)	0 (0%)	7(3.4%)	0.003*
Bleeding requiring surgery, n(%)	0 (0%)	3(1.5%)	0.079*
Urethral stricture, n(%)	0 (0%)	1(0.5%)	0.430*
Postoperative recurrence requiring reoperation#, n(%)	0 (0%)	3(1.5%)	0.079*
Grade IV, n(%)	0 (0%)	0 (0%)	
Grade V, n(%)	0 (0%)	0 (0%)	
**Total, n(%)**	**19 (7.0%)**	**44(21.6%)**	**<0.001**

**B-TUERP** = bipolar transurethral enucleation and resection of the prostate; **B-TURP** = bipolar transurethral resection of the prostate; **TURS** = transurethral resection syndrome.

* Fisher's exact test; #due to inadequate resection in the first procedure

## DISCUSSION

In recent years, B-TURP has been advocated as an alternative to monopolar TURP-the gold standard for the surgical treatment of BPH (19). However, since the B-TURP technique is not substantially different from the monopolar technique and the amount of resected prostatic tissue did not differ significantly between the two procedures, the functional results of B-TURP are similar to those of monopolar TURP (20). A meta-analysis of 16 randomized, controlled-trials involving 1406 patients showed no clinically relevant difference in short-term efficacy between monopolar and BTURP procedures (21). In contrast, the TUERP technique replicates the open enucleation of prostatic adenomas in an endoscopic fashion and combines the benefits of complete enucleation and a minimally invasive approach to BPH (16), allowing for more complete adenoma removal. We therefore in the present study compared the efficacy and safety of B-TUERP versus B-TURP in the management of prostates larger than 60g. Unsurprisingly, we found that when compared with the B-TURP procedure, BTUERP was associated with shorter operative time, postoperative bladder irrigation duration and hospital stay. Furthermore, there was a greater weight of resected prostatic tissue, less postoperative hemoglobin decrease, better postoperative IPSS and Qmax, and lower incidences of hyponatremia, urinary sepsis, blood transfusion requirement and reoperation. All these suggest that B-TUERP is safe and feasible in the treatment of prostates larger than 60g.

After the adenoma was detached from the surgical capsule during TUERP, the blood supply to the adenoma was cut off and hemostasis was performed by coagulation under endoscopic monitoring (12). Therefore, the resection of the detached adenoma is virtually bloodless (15). In contrast, during TURP the vessels are repeatedly cut until the surgical capsule is reached (16). Therefore, intraoperative blood loss will be less in the B-TUERP procedure than in the B-TURP procedure (12, 16). Consistent with this previous observation, we found that postoperative hemoglobin decrease and the numbers of patients requiring blood transfusion and those developing bleeding requiring surgery differed significantly in favor of the TUERP procedure. Due to improved operative field visibility, decreased capsular perforation and more rapid, complete tissue removal (16), the operative time, postoperative bladder irrigation duration and hospital stay were significantly shortened in the TUERP procedure compared with the TURP procedure.

Excessive intraoperative absorption of irrigation fluid may lead to the occurrence of TURS, and the use of saline for irrigation can reduce the fluid absorption-associated morbidity and eliminate the risk of TURS (22, 23). In the current study, no TURS occurred in either the B-TURP group or the BTUERP group, because both procedures used normal saline as irrigant. However, we found that the incidence of hyponatremia was significantly higher in the B-TURP group than in the B-TUERP group. This discrepancy may be explained by longer operative time and greater intraoperative blood loss associated with the B-TURP procedure.

Since BPH patients often develop urinary retention and urinary tract infections, bacteria in urine can spread via blood vessels or perforated prostatic capsule and induce urinary sepsis (24). When the adenoma is enucleated during B-TUERP, hemostasis is performed by coagulation. Thus, the chance of prostatic capsular perforation and the incidence of uri\nary sepsis are greatly reduced. In the present study, four patients in the B-TURP group developed urinary sepsis, whereas no patients in the B-TUERP group developed this complication.

Studies have shown that the incidences of urethral stricture and bladder neck stenosis are not different significantly between the bipolar and monopolar TURP procedures (7). In this study, we found that the incidences of urethral stricture, postoperative acute urinary retention and bladder neck stenosis did not differ significantly between the bipolar TUERP and TURP procedures, suggesting that resection type is not a significant predictor of the risk of these complications.

Ideal TURP should involve accurate, complete removal of the adenoma. However, when performing traditional TURP, it is difficult to accurately judge the boundary between outer and inner glands and the depth of excision. This often results in excessive resection which may induce capsular perforation, or results in insufficient removal of the adenoma (18). Particularly, when the volume of the prostate gland is large, e.g., significantly above the level of the verumontanum, the surgeon often does not cut enough prostatic tissue at the apex due to serious concern about damaging the urethral sphincter and causing incontinence (12). As a result, recurrence often develops. Since the B-TUERP allows the removal of the adenoma accurately and completely (12, 15, 16), there is often little residual hyperplasia tissue. Unsurprisingly, although four patients in the B-TURP group needed reoperation during the 2-year follow-up period, no patients in the B-TUERP group required reoperation because of recurrence.

Our study has several limitations. The non-randomized retrospective nature of the study is associated with a high risk of bias and may influence the interpretation of our data. In this single-center study, the relatively small sample size and short follow-up duration might lead to low statistical power and limit the strength of our conclusions. Furthermore, the inability to measure intraoperative blood loss and postoperative PV is another limitation of our study. Due to the resected cavity, the size of the residual adenoma cannot be exactly measured. Larger studies conducted in multiple centers will be required in future to confirm the findings of the present study.

In conclusion, our findings suggest that BTUERP is superior to B-TURP in the management of prostates larger than 60g in terms of shorter operative time, postoperative bladder irrigation duration and hospital stay. There is also a greater weight of resected prostatic tissue, less postoperative hemoglobin decrease, better postoperative IPSS and Qmax. There are lower incidences of hyponatremia, bleeding, urinary sepsis, blood transfusion requirement, transitory incontinence and reoperation. However, longer-term and larger studies are needed to validate these results.

## ABBREVIATIONS

BPH: = benign prostatic hyperplasia
B-TUERP: = bipolar transurethral enucleation and re-section of the prostate
B-TURP: = bipolar transurethral resection of the prostate
IPSS: = International Prostate Symptom Score
PV: = prostatic volume
Qmax: = maximal urinary flow rate
QoL: = quality of life
RUV: = residual urine volume
TPSA: = total prostate specific antigen
TUERP: = transurethral enucleation and resection of the prostate
TURP: = transurethral resection of the prostate
TURS: = transurethral resection syndrome
